# Home care in COVID-19 patients with the home-quarantined condition: A study from Iran

**DOI:** 10.3389/fpubh.2022.952618

**Published:** 2022-09-06

**Authors:** Poorandokht Afshari, Maryam Beheshti-Nasab, Elham Maraghi, Simin Sadeghi, Nafiseh Sanjari, Kourosh Zarea

**Affiliations:** ^1^Reproductive Health Promotion Research Center, Ahvaz Jundishapur University of Medical Sciences, Ahvaz, Iran; ^2^Nursing and Midwifery Faculty, Ahvaz Jundishapur University of Medical Sciences, Ahvaz, Iran; ^3^Department of Biostatistics and Epidemiology, Ahvaz Jundishapur University of Medical Sciences, Ahvaz, Iran; ^4^Imam Khomeini Hospital, Ahvaz Jundishapur University of Medical Sciences, Ahvaz, Iran; ^5^Arak University of Medical Sciences, Arak, Iran; ^6^Nursing Care Research Center in Chronic Diseases, Ahvaz Jundishapur University of Medical Sciences, Ahvaz, Iran

**Keywords:** COVID-19, self-care, family care, home care, home-quarantined, home isolation

## Abstract

**Objectives:**

During the COVID-19 home-quarantines, home care services may act as an auxiliary component of health care system, which reduces the burden on the formal health care system. This study aimed to investigate the status of informal home care provided for home quarantined patients with COVID-19 in southwest Iran.

**Methods:**

This descriptive study was conducted on 288 patients with COVID-19 in Ahvaz (southwest Iran). Inclusion criteria included a known case of COVID-19, willingness to participate in the study, recommendation of home quarantine from a health center, having a smartphone, and fluency in reading and writing in Persian, and the exclusion criterion was a history of COVID-19 longer than 3 months. The data collection method was structured interviews based on a questionnaire (face-to-face-telephone calls-video call). SPSS software was used for the analysis of data.

**Results:**

45.5% of the participants in the study were women with a mean age of 37.82 (10.48%) and 55.5% were men with a mean age of 36.12 (11.93%). Findings showed that in most cases, the spouse (61.4) is responsible for the care of the patient, and in other cases, parents are responsible for this duty. 57.3% of the patients stated that they themselves had to leave home to provide for necessities of life, and 37.2% stated that they were in charge of cooking. 47.9% of the patients evaluated the quality of quality of care provided at home as good. Most of patients and caregivers referred to hospital for getting information (35.8% patients and 34% caregivers). Most of patients recovered from diseases (60.8%) and 39.2% were hospitalized. Although 43.9% of men and 33.6% of women were hospitalized and a there was a significant difference between men and women (*P* < 0.04).

**Conclusion:**

During COVID-19 pandemic home care to reduce the burden on the health system are very important. We must also know that this type of care requires informed and planned support and sufficient community education. The health care system needs to put self-care and family care among its top priorities. The focus should be on educational and mental support of informal caregivers along with measures that protect their relatives from COVID-19.

## Introduction

COVID-19 is a highly contagious disease that is transmitted from one person to another through droplets and contact. Symptoms usually include fever, dry cough, myalgia, and shortness of breath. The severity of the disease can range from mild to severe to critical (1). During this pandemic, many nurses have found innovative and creative ways to involve families in the care of the patient by using the new technology to create a good connection between family members and the patient, and in addition to helping the patient, care is provided to the family members of these patients (2). The experience of caring for patients with SARS shows that the evaluation and care of these patients are costly and require a lot of facilities and manpower. In addition, providing care increases the risk of transmitting the disease to other patients, nurses, and medical staff (3).

During the COVID-19 home quarantines, home care services may act as an auxiliary component of health care services, which reduces the burden on the formal health care system (4). Home quarantine not only prevents various infectious diseases but also reduces the cost of hospitalization (5). Patient care at home has received momentum with the global increase in the elderly population, and family care has become an important component of long-term care. In this respect, women have been given this responsibility as the main family caregivers (6). Most people prefer to receive care services in their homes where they live with their family members (7). Home care is an aspect of care continuity that can be effective in facilitating the treatment process of patients who do not need to be hospitalized and their care needs are managed and followed up at home (8). Approximately 81% of patients with COVID-19 show mild symptoms and recover at home; therefore, quarantine and home care are some of the common treatments for this disease (9). In India, during the COVID-19 epidemic, the provision of health care was challenged by major problems such as a large number of patients, the fatigue of health care workers, and the lack of vital medical equipment such as ventilators and oxygen. Virtual COVID In-Patient (VCIP) care at home, sleep and exercise counseling, and even medication through video counseling and door-to-door laboratory facilities helped the treatment system. This was confirmed by the 99.5% decrease in the rate of hospitalization and patients' satisfaction with their care experience (10). According to the Centers for Disease Control (CDC) and Prevention, to care for a patient with COVID-19 at home, it is essential to help meet basic needs, help the patients follow the doctor's instructions, and make sure they take the prescribed medications. The caregivers should also ensure that the patients drink plenty of fluids and rest sufficiently (11). With respect to caring for COVID-19 patients, Safari highlights the importance of personal hygiene, using gauze and masks during care, hand washing, and proper disinfection of the environment and equipment (12).

It should be noted that during the COVID-19 outbreak, more attention was paid to hospital care issues, and the challenges of home care services were overlooked. Although COVID-19 patient care shifts at home are longer, less attention is paid to the physical, mental, and social health of home care providers. Some reports point to environmental challenges in caring for COVID-19 patients at home. For example, these patients should be cared for in a separate, well-ventilated room, which may not be possible for all patients due to limited space or a large number of family members present at home. This in turn leads to infection control challenges for patients and their families (4). Findings from a cross-sectional survey in a Hong Kong urban area affected by the COVID-19 pandemic showed that the double burden of primary care for COVID patients is imposed on many informal health care providers. Some of these informal home care providers do not have sufficient knowledge about care tasks (13). It seems that during the epidemic of COVID-19, most of the attention is directed toward the problems of hospital care, and the challenges of home care services have been paid less attention (14). Adiukwu et al. believe task shifting to no specialist health workers to use of the available resources and technology has become the most viable strategy (15). Home care providers can serve as a supplemental component of healthcare services and significantly lighten the load on the health system. However, very little is known about the difficulties faced by informal home care providers during the COVID-19 pandemic (16). It can be beneficial to improve health care to pay particular attention to the difficulties and issues associated with home care, particularly during times of crisis (17). In Iran, as in all parts of the world, many patients with COVID-19 receive home care often by their family members, especially during novel corona pandemic rush hours. This study aimed to investigate the status and condition of care provided by informal home care providers for patients with COVID-19.

## Materials and methods

### Study design

This descriptive study was conducted to investigate the home care provided to patients with COVID-19.

### Population (inclusion and exclusion criteria)

The study population included all men and women with COVID-19 in Ahvaz. Three hundred forty people were randomly selected from a list of 1,600 people provided by the Ahvaz Vice-Chancellor for Health. Inclusion criteria were: a known case of COVID-19, willingness to participate in the study, recommendation of home quarantine from a health center, having a smartphone, and fluency in reading and writing in Persian, and the exclusion criterion was a history of COVID-19 longer than 3 months due to the possibility of recall bias.

### Process of study

To start the research, the administrative steps included ethical confirmation and formal letters were taken. Three hundred forty people were randomly selected from a list of 1,600 people with COVID-19. By making telephone calls, the eligible individuals were identified. They were briefed on the objectives of the research, and they are informed to complete the questionnaire consent was obtained. Then, if the person was willing, a video call would be made through WhatsApp at the same time, and the interview would be conducted based on the available questionnaire. Otherwise, a voice interview would be conducted on phone. In cases where the callees declared that they were not ready to answer the questions at the moment, the date and time of the next interview were arranged with them. Out of the 340 eligible participants, 288 fully answered the questions and completed the questionnaire.

### Research instruments

The data collection method was structured interviews based on a questionnaire. Four steps were taken to prepare the data collection tool which was a researcher-made questionnaire. First, an initial questionnaire was developed by studying the related literature by the research team. This questionnaire was then given to eight faculty members of the School of Nursing and Midwifery to verify its content validity (CVI and CVR). The third stage was piloting the questionnaire on 15 available patients (colleagues who were infected and not hospitalized) in order to determine the reliability of the questionnaire, and a Cronbach's alpha of 0.84 was obtained. The final step was designed in 5 different sections. The first section dealt with the demographic information (5 questions), the second section was related to corona infection in the patient and the people who lived with him (10 questions), and the third section was concerned with how to observe hygiene at home and how home quarantine is done (10 questions), the fourth section was about how to follow up care in health centers (5 questions), and the last section of the questionnaire was devoted to the type of care provided at home (7 questions).

### Statistical analysis

Continuous variables are reported as mean±StD. Categorical data are expressed as numbers (percentages). The normality of continuous variables was examined using Shapiro-Wilk's W-test. Two independent samples *t*-test was used to compare the mean age between men and women. Categorical variables were compared using the Chi-square test. Statistical analysis was performed using the statistical software SPSS 18.0.0. (SPSS Inc. Chicago, IL, USA). A *P*-value of < 0.05 was considered to indicate statistical significance for all analyses.

## Results

Analysis of data from 288 patients showed that 45.5% of the participants in the study were women with a mean age of (37.82 ± 10.48) and 55.5% were men with a mean age of (36.12 ± 11.93%). Most participants were government employees. Most of the participants had no underlying disease (51%) and the disease duration was 21–30 days. There was not any difference between men and women in demographic variables and clinical characteristics (Table 1).

**Table 1:** Demographic and clinical characteristics of COVID-19 patients with the home-quarantined condition.

**Variables**	**All participants** **(*n* = 288)**	**Male** **(*n* = 157)**	**Female** **(*n* = 131)**	***P***
***Demographic***				
Age group; *n* (%)				0.169
<20	9 (3.1)	6 (3.8)	3 (2.3)	
20–29	80 (27.8)	50 (31.8)	30 (22.9)	
30–39	96 (33.3)	50 (31.8)	46 (35.1)	
40–49	55 (19.1)	24 (15.3)	31 (23.7)	
50+	48 (16.7)	27 (17.2)	21 (16)	
*Mean ± St.d*	36.87 ± 11.3	36.12 ± 11.03	37.82 ± 10.48	
Occupation; *n* (%)				
Housewife	51 (17.8)	-	51 (17.8)	-
Jobless	32 (11.1)	32 (11.1)	-	
Self-employed	53 (18.42)	40 (13.9)	13 (4.52)	
Governmental-employee	126 (52.77)	67 (32.27)	59 (20.5)	
Education; *n* (%)				
Primary school	40 (13.9)	24 (8.3)	16 (5.6)	
High school	65 (22.5)	38 (13.1)	27 (9.4)	
University degree	183 (63.6)	95 (33)	88 (30.6)	
Marital status; *n* (%)				0.052
Single	113 (39.2)	70 (44.6)	43 (32.8)	
Married	175 (60.8)	87 (55.4)	88 (67.2)	
Number of family members; *n* (%)				-
1–3	132 (45.8)			
4–5	114 (39.6)			
6–7	42 (14.6)			
Underlying disease; *n* (%)				0.847
No disease	147 (51)	80 (51)	67 (51)	
Diabetes	24 (8.3)	16 (10.2)	8 (6.1)	
HTN	21 (7.3)	12 (7.6)	9 (6.9)	
CVD	21 (7.3)	11 (7)	10 (7.6)	
Mental disease	15 (5.2)	8 (5.1)	7 (5.3)	
Respiratory disease	23 (8)	11 (7)	12 (9.2)	
HTN and diabetes	18 (6.3)	8 (5.1)	10 (7.6)	
HTN and respiratory disease	5 (1.7)	4 (2.5)	1 (0.8)	
Respiratory disease and CVD	14 (4.9)	7 (4.5)	7 (5.3)	
Disease duration; *n* (%)				0.716
1–20 days	124 (43.1)	71 (45.2)	53 (40.5)	
21–30 days	126 (43.8)	64 (40.8)	62 (47.3)	
31+ days	38 (13.2)	22 (14)	16 (12.2)	

Most patients were aware of how getting infected with the disease, and about 50% of the cause of the disease was participation in social gatherings such as ceremonies and funerals (even in some cases, the funeral of somebody who had died from COVID-19) and weddings, formal and informal meetings, and gatherings such as those in parks and shopping malls. In most cases, at least one family member was infected at the same time as the patient had the disease, and there was a history of simultaneous or previous infection in family members interacting with the patient. A comparison of hygiene protocols observed before and after recovery shows that before recovery, the greatest emphasis was on washing and disinfecting surfaces, and many used only masks (23.6%), but after recovery, several protocols were observed, including hand washing, and the simultaneous use of the mask had increased. Unfortunately, the important point was that social distancing was observed neither before nor after the infection was zero. There was not any difference between men and women in the disease's contamination and hygiene observance (Table 2).

**Table 2:** Type of disease contamination and the degree of hygiene observance before disease and during home quarantine.

**Variables**	**All participants** **(*n* = 288)**	**Male** **(*n* = 157)**	**Female** **(*n* = 131)**	***P***
Contamination method; *n* (%)				0.72
Family	4 (1.4)	1 (0.6)	3 (2.3)	
At work	33 (11.5)	15 (9.6)	18 (13.7)	
Social gatherings	142 (49.4)	85 (54.1)	57 (43.5)	
Not wearing mask	58 (20.1)	29 (18.5)	29 (22.1)	
Unknown	51 (17.7)	27 (17.2)	24 (18.3)	
Simultaneous infection; *n* (%)				0.29
No one	28 (9.7)	15 (9.6)	13 (9.9)	
Spouse	94 (32.7)	50 (31.8)	44 (33.6)	
Parents	77 (26.7)	48 (30.6)	29 (22.1)	
All family members	89 (30.9)	44 (28)	45 (34.4)	
Testing the rest of the family members after one member is diagnosed with the disease; *n* (%)				0.28
Yes	128 (44.4)	65 (41.4)	63 (48.1)	
No	160 (55.6)	68 (58.6)	92 (51.9)	
The current status of the infected person in the family; *n* (%)				0.854
Recovered	175 (60.8)	93 (59.2)	82 (62.6)	
Deceased	23 (8)	14 (8.9)	9 (6.9)	
Recovered/Deceased	34 (11.8)	20 (12.7)	14 (10.7)	
Not infected	56 (19.4)	30 (19.1)	26 (19.8)	
The degree of hygiene observance before disease; *n* (%)				0.12
Disinfecting hands and surfaces	92 (31.9)	49 (32.2)	43 (32.3)	
Wearing masks	68 (23.6)	40 (26.5)	28 (21.1)	
Face steaming and gargling with salt water	4 (1.4)	1 (0.6)	3 (2.3)	
Wearing masks and washing hands	70 (24.3)	34 (22.6)	36 (26.2)	
All of the above	14 (4.9)	4 (3.5)	10 (7.3)	
Social distancing	0 (0)	0 (0.0)	0 (0.0)	
No action	40 (13.9)	22 (14.6)	18 (12.3)	
The degree of hygiene observance after getting infected; *n* (%)				0.13
Disinfecting hands and surfaces with alcohol	116 (40.3)	50 (31.8)	66	
Wearing masks	4 (1.4)	1	3	
Hand washing, face steaming, and gargling with salt water	36 (12.5)	16	20	
Wearing masks and washing hands	89 (30.9)	41	37	
All of the above	43 (14.9)	21	22	
Social distancing	0 (0)	0	0	
No action taken	0 (0)	0	0	

Table 3 shows the role of health centers in supervising and monitoring the patient's condition at home. As evident in this table, it can be observed that despite telephone follow-ups and even in-person follow up in 52.8 percent of health centers, unfortunately, many others (47.2) did not follow up on the condition of the patients at home (Table 3).

**Table 3:** Health centers' supervision and guidance concerning the patient's condition at home.

**Variables**	**All participants** **(*n* = 288)**	**Male** **(*n* = 157)**	**Female** **(*n* = 131)**	***P***
Distributing brochures containing hygiene tips at home; *n* (%)				0.23
Yes	133 (46)	76 (48.4)	57 (43.5)	
No	155 (54)	74 (51.6)	81 (45.5)	
Length of quarantine as recommended by health centers; *n* (%)				0.610
2 weeks	171 (59.4)	97 (61.8)	74 (56.5)	
<2 weeks	41 (14.2)	20 (12.7)	21 (16)	
More than 2 weeks	76 (26.4)	40 (25.5)	36 (27.5)	
Health centers' self-care recommendations to the patient; *n* (%)				0.345
Careful use of medications	10 (3.5)	8 (2.5)	4 (3.1)	
Careful use of medications and observing personal hygiene	71 (24.6)	36 (21.6)	35 (26.7)	
Careful use of medication and eating the right foods	88 (30.6)	53 (28.6)	36 (27.2)	
Observing personal and environmental hygiene	25 (8.7)	13 (7.2)	14 (10.7)	
All of the above	94 (32.6)	53 (28.6)	42 (32.3)	
Health centers' follow-up of the home-quarantined patient's condition; *n* (%)				0.474
Phone call	98 (34)	57 (36.3)	41 (31.3)	
Visiting at home	54 (18.8)	31 (19.7)	23 (17.6)	
No follow-up	136 (47.2)	69 (43.9)	67 (51.1)	

Our findings about how to provide home care to patients with COVID-19 showed that in most cases, the spouse (61.4%) is responsible for the care of the patient, and in other cases, parents are responsible for this duty. Interestingly, 57.3% of the patients stated that they themselves had to leave home to provide necessities of life, and 37.2% stated that they were in charge of cooking. As with the quality of home care, 47.9% of the patients evaluated the quality of self-care provided at home as good. The final status of patients showed that 60.8% of participants recovered from diseases. In comparison between males and females was revealed that males were more hospitalized (43.9%) than females (33.6%) and Fisher exact test showed a significant difference (*P* < 0.04) (Table 4).

**Table 4:** Patient evaluation of the type of care provided during home quarantine.

**Variables**	**All participants** **(*n* = 288)**	**Male** **(*n* = 157)**	**Female** **(*n* = 131)**	***P***
Caregiver at home; *n* (%)				0.65
Spouse	177 (61.4)	88 (56.7)	87 (66.4)	
Parents	61 (21.2)	41 (26.1)	21 (16.1)	
Siblings	15 (5.2)	8 (5.1)	7 (5.3)	
No caregiver (the patients themselves were the caregivers)	35 (11.11)	19 (12.1)	16 (12.2)	
Quality of care provided at home from the patient's perspective; *n* (%)		
Good	138 (47.9)	75 (47.8)	63 (48.1)	0.52
Moderate	150 (52.1)	82 (52.2)	68 (51.9)	
Caregiver's educational attainment; *n* (%)				0.082
Related to medical sciences	36 (12.5)	24 (15.3)	12 (9.2)	
Not related to medical science	252 (87.5)	133 (84.7)	119 (90.8)	
The way patients deal with a question or problem; *n* (%)				0.96
Patient's referring to the hospital	103 (35.8)	52 (33.1)	51 (38.9)	
Caregiver's referring to hospital	98 (34)	62 (39.5)	36 (27.5)	
Making phone calls to health centers	87 (30.2)	43 (27.4)	44 (33.6)	
Shopping by the patient during bed rest; *n* (%)				0.188
Shopping: Yes	165 (57.3)	84 (53.5)	81 (61.8)	
Shopping: No	123 (42.7)	73 (46.5)	50 (38.2)	
Cooking by the patient during bed rest; *n* (%)				0.142
Cooking: Yes	107 (37.2)	52 (33.1)	55 (42)	
Cooking: No	181 (62.8)	105 (66.9)	76 (58)	
Final status of patient in home quarantine, *n* (%)				0.047*
Recovered	175 (60.8)	88 (51.6)	87 (66.4)	
Hospitalized	113 (39.2)	69 (43.9)	44 (33.6)	

*P < 0.05.

## Discussion

This study aimed to investigate the type of care provided to home-quarantined patients with COVID-19 and the people providing this care.

Clinical characteristics of patients showed that most of them had no underlying diseases and duration of their diseases had taken about 21 to 30 days. This findings is same as international studies (9, 18). In the present study, we inquired about how patients had got infected, and the results showed that most of them were infected due to their participation in large gatherings, and attending shopping malls and parks, hence spreading the disease to their family members. The participants in our study said that despite the recovery of most patients, the involvement of some of these patients with the disease led to death, and 27.1% of the same statistical population did not consider their physical condition normal even after the end of their illness and believed that the disease had affected their health. Comparing the way hygiene protocols were observed before and after recovery showed that before the infection, many people followed no hygiene protocols, and some limited these protocols to washing and disinfecting hands or surfaces or only wearing masks. However, after the infection, people tended to use several protocols at the same time, and the participants engaged in at least one preventative behavior. Interestingly, however, social distancing as one of the most basic health protocols to avoid getting COVID-19 was ignored totally. It seems that this problem should be addressed by some kind of consciousness-raising interventions or passing government laws and regulations. According to Ramezani et al., since the main method of this virus is transmitted through person-to-person contact, frequent hand washing, maintaining social distance, and personal hygiene play an important role in preventing the disease (19). Results of China's experience with new coronavirus pneumonia have shown that social distancing is the most effective way to control the disease (20). Governments have implemented social distancing measures to combat the COVID-19 pandemic. This includes a set of instructions that people need to follow when attending a gathering (21). According to Pedersen and Favero, social distancing as an effective means of curbing the outbreak of COVID-19 but believes that social distancing is appropriate provided that all people participate in it. Compared with the public, government officials have a more decisive role in this regard, and they should try to make the public aware of the importance of social distancing and persuade them to observe this preventative measure. Where people are less engaged in social distancing or are less likely to engage in severe social distancing, mass media such as television and radio should provide community-level education (22).

Many patients receiving care at home are not followed up by the health center, and in case they have any problems or questions, they or a relative of theirs should go to the hospital for this, and the only instruction offered to these families is to provide this demanding and important type of care was a brochure including a general overview of COVID-19. According to some studies conducted in Iran, home care givers is faced with problems including environmental challenges, problems related to insurance, lack of knowledge, and a lack of personal protective equipment (4, 14). Many researchers believe that the hospitalized patient should not be left alone and should be monitored by the hospital and the health system. In India, Virtual COVID In-Patient (VCIP) care at home was offered to prevent hospitalization by providing sleep and exercise counseling, and even medication through video counseling and door-to-door laboratory facilities (10). The World Health Organization recommends that in cases where care should be provided at home, the nurse should assess whether or not the home environment is appropriate for continued care. The health care professional should assess whether the patients and their family members can adhere to the recommended precautions (hand sanitation, healthy respiratory conditions, clean environment, and mobility restrictions inside or outside the home) (23). A relation needs to be established and maintained with the nurse or health team, or both, during the patient's stay at home, i.e., until the patient's symptoms have completely disappeared. Patients and family members should be informed about personal hygiene, safety protocols, primary prevention, and infection control measures so that they can safely care for a person suspected of having COVID-19 in order to prevent the transmission of the infection to other family members (24, 25).

Results of the present study indicated that care was provided by first-degree family members such as spouse, mother, father, and siblings during quarantine. However, several family members often contaminated with disease, and patients with COVID-19 had to break the quarantine and leave the house to buy the necessities of life or do the cooking and housework themselves. Therefore, in many cases, the way care is provided to the patient seems to be different from the concept of care provision in the health care system. Patient care refers to the prevention, treatment, and management of disease and maintenance of physical and mental health through the services provided by health professionals or non-professionals under their supervision (26). The current epidemic crisis can potentially affect the provision of quality nursing care, resulting in the loss and endangering of nursing care (27). Certainly, home care, whether for chronic diseases or during a pandemic that suddenly imposes a heavy burden on the healthcare system, will be a tremendous help to the healthcare system and will take a considerable part of this burden off the healthcare system, especially with respect to treatment, enabling this system to provide services more energetically and with better quality. However, two important points should be borne in mind: First, caregivers need to raise awareness about how to care for a patient far more comprehensive than what is simply put on a brochure, as research findings in Hong Kong during the COVID-19 pandemic show, the caregivers responsible for providing care to patients do not have sufficient knowledge of the care duties (16). Therefore, raising the awareness of the caregiver through public media can be well implemented. Telemedicine is one of the most important technological innovations of the late twentieth century which serves as a gateway to modern health care. The goal of telemedicine was to improve the quality of care, enhance patient safety, and provide rapid access to health care by overcoming geographical barriers. The use of telephones, cell phones, text messages, and communication technologies is part of distance nursing which is one form of telemedicine (3, 8). The second issue is that caregivers at home should be helped to create the appropriate environment. These caregivers do not have hospital equipment, supplies, and protection, and may have physical and mental problems. The WHO, recommends that in cases where home care is to be provided, the trained health system should, if possible, assess whether the residential environment is suitable for care or not or whether any precautions are recommended as part of home care (24).

The health system should make an initial assessment in dealing with the patient and family according to the individual characteristics of the patient and caregivers, and if necessary, create a home visit team to include these assessments in its work plan. There is a great need within this population for interventions, which effectively reduce the burden diseases (28). Vadivel believe that after the pandemic's peak, there may be an increase in the prevalence of mental health problems among the at-risk population and those who have risk factors (29). Policymakers still need to work hard to educate the public adequately about adopting COVID-19 safety behaviors (24).

One of current study finding was revealed that men with COVID-19 were more hospitalized than women. This finding is in line with Jin et al. that founded the number of men was 2.4 times that of women in the deceased patients. While men and women had the same susceptibility, men were more prone to dying. Men with COVID-19 are more at risk for worse outcomes and death, independent of age (30).

The present study has some limitations. Firstly, this study is cross-sectional in nature, and caregiver stress during data gathering may have affected the response of participants. Second, this study was only conducted in southwest of Iran; however, the COVID-19 situation varies by country and region. Therefore, further research is needed to generalize our findings to other countries.

## Conclusion

Our findings showed that situation and type of informal homecare in the COVID-19 pandemic in south of Iran. The COVID-19 pandemic will end and human beings will pay a high price for the lessons they learned from it. One of the most important lessons is to value self-care and family care to reduce the burden of the health system. However, we must also know that this type of care requires informed and planned support and sufficient community education. The health care system needs to put self-care and family care among its top priorities. The focus should be on educational and mental supporting the participation of the caregivers along with measures that protect the caregivers and their relatives from COVID-19. Our results lead to the implications that all institutions with a mandate to support informal caregivers be provided with education and support them in order to be able to protect others and themselves and to continue their work throughout the pandemic.

## Data availability statement

The datasets generated during and/or analyzed during the current study will be available from the corresponding author on reasonable request.

## Ethics statement

The studies involving human participants were reviewed and approved by Ahvaz Jundishapur University of Medical Sciences with code: IR.AJUMS.REC.1399.405. The patients/participants provided their written informed consent to participate in this study.

## Author contributions

PA and KZ conceived of the presented idea. PA and MB-N developed the theory and performed the computations and investigated and supervised the findings of this work. EM verified the analytical methods. SS and NS supervised all of the parts of the data collection. All authors contributed to the article and approved the submitted version.

## Funding

This project was approved by the Vice-Chancellor for Research of Ahvaz Jundishapur University of Medical Sciences (Ref. NCRCCD-9912).

## Conflict of interest

The authors declare that the research was conducted in the absence of any commercial or financial relationships that could be construed as a potential conflict of interest.

## Publisher's note

All claims expressed in this article are solely those of the authors and do not necessarily represent those of their affiliated organizations, or those of the publisher, the editors and the reviewers. Any product that may be evaluated in this article, or claim that may be made by its manufacturer, is not guaranteed or endorsed by the publisher.

## References

[1] Sharma SK Nuttall C Kalyani V. Clinical nursing care guidance for management of patient with COVID-19. J Pak Med Assoc. (2020) 70(Suppl. 3):S118–23. 10.5455/JPMA.29 32515397

[2] Luttik MLA Mahrer-Imhof R García-Vivar C Brødsgaard A Dieperink KB Imhof L. The COVID-19 pandemic: a family affair. J Fam Nurs. (2020) 26:87–9. 10.1177/1074840720920883 32427038 PMC7265214

[3] Purabdollah M Ghasempour M. Tele-nursing new opportunity for nursing care in covid-19 pandemic crisis. Iran J Public Health. (2020) 49(Suppl 1):130–1. 10.18502/ijph.v49iS1.3685 34268221 PMC8266018

[4] Keyvanloo Shahrestanaki S. Challenges of Home Care during the COVID-19 Outbreak. Iran. J. Nurs. Res. (2020) 33:1–6. 10.29252/ijn.33.127.1

[5] Gholipour B Bigli S Sadat Z Forooshani D Bayat ZS Montazer B. Research paper effect of telenursing education on the comfort of patients with COVID-19 in home quarantine. J Mod Fam Med. (2021) 1:2–13. 10.32598/JFM.1.1.102

[6] Amini R Shahboulaghi FM Tabrizi KN Forouzan AS. Facilitators and barriers to social participation of community-dwelling older adults in Iran: a qualitative study. Iran J Ageing. (2021) 16:172–87. 10.32598/sija.16.2.3052.1

[7] Heydari H. Home-based palliative care: a missing link to patients' care in Iran. Hayat. (2018) 24:97–101. Available online at: https://hayat.tums.ac.ir//article-1-2281-en.html

[8] Kord Z Fereidouni Z Mirzaee MS Alizadeh Z Behnammoghadam M Rezaei M. Telenursing home care and COVID-19: a qualitative study. BMJ Support Palliat Care. (2021). 10.1136/bmjspcare-2021-003001. [Epub ahead of print]. 34187878

[9] Jutzeler CR Bourguignon L Weis CV Tong B Wong C Rieck B. Comorbidities, clinical signs and symptoms, laboratory findings, imaging features, treatment strategies, and outcomes in adult and pediatric patients with COVID-19: a systematic review and meta-analysis. Travel Med Infect Dis. (2020) 37:101825. 10.1016/j.tmaid.2020.101825 32763496 PMC7402237

[10] Kesavadev J Basanth A Krishnan G Vitale R Parameswaran H Shijin S. A new interventional home care model for COVID management: virtual Covid I. Diabetes Metab Syndr. (2021) 15:102228. 10.1016/j.dsx.2021.102228 34330071 PMC8299213

[11] CDC. COVID19-Caring for Someone at Home. Center for Disease Control and Prevention (2021). Available online at: https://www.cdc.gov/coronavirus/2019-ncov/if-you-are-sick/care-for-someone.html (accessed May 24, 2022).

[12] Saffari M Vahedian-Azimi A Mahmoudi H. Nurses' experiences on self-protection when caring for COVID-19 patients. J Mil Med. (2020) 22:570–9. 10.30491/JMM.22.6.57033274422

[13] Hung KKC Walline JH Chan EYY Huang Z Lo ESK Yeoh EK. Health service utilization in Hong Kong During the COVID-19 pandemic–a cross-sectional public survey. Int J Health Policy Manag. (2020) 11:508–13. 10.34172/ijhpm.2020.183 33105965 PMC9309937

[14] Raoofi A Takian A Sari AA Olyaeemanesh A Haghighi H Aarabi M. COVID-19 pandemic and comparative health policy learning in Iran. Arch Iran Med. (2020) 23:220–34. 10.34172/aim.2020.02 32271594

[15] Adiukwu F de Filippis R Orsolini L Gashi Bytyçi D Shoib S Ransing R. Scaling up global mental health services during the COVID-19 pandemic and beyond. Psychiatr Serv. (2022) 73:231–4. 10.1176/appi.ps.202000774 34235945

[16] Chan EYY Gobat N Kim JH Newnham EA Huang Z Hung H. Informal home care providers: the forgotten health-care workers during the COVID-19 pandemic. Lancet. (2020) 395:1957–9. 10.1016/S0140-6736(20)31254-X 32497509 PMC7263813

[17] Brant JM Fink RM Thompson C Li YH Rassouli M Majima T. Global survey of the roles, satisfaction, and barriers of home health care nurses on the provision of palliative care. J Palliat Med. (2019) 22:945–60. 10.1089/jpm.2018.0566 31380727

[18] WHO. Report of the WHO-China Joint Mission on Coronavirus Disease 2019 (COVID-19) (16–24 February 2020). p. 1–40. Available online at: https://www.who.int/docs/default-source/coronaviruse/who-china-joint-mission-on-covid-19-final-report.pdf

[19] Ramezani A Amirpour M. Nutritional care in the prevention and treatment of coronavirus disease 2019: a simple overview. J Health Res Commun. (2020) 6:74–82. Available online at: http://jhc.mazums.ac.ir/article-1-476-en.html

[20] Qian M Jiang J. COVID-19 and social distancing. J Public Health. (2022) 30: 259–61. 10.1007/s10389-020-01321-z 32837835 PMC7247774

[21] Mohler G Bertozzi AL Carter J Short MB Sledge D Tita GE. Impact of social distancing during COVID-19 pandemic on crime in Los Angeles and Indianapolis. J Crim Justice. (2020) 68:101692. 10.1016/j.jcrimjus.2020.101692 32501302 PMC7252124

[22] Pedersen MJ Favero N. Social distancing during the COVID-19 pandemic: who are the present and future noncompliers? Public Adm Rev. (2020) 80:805–14. 10.1111/puar.13240 32836442 PMC7280647

[23] World Health Organization. Home Care For Patients With Suspected or Confirmed COVID-19 and Management of their Contacts. (2020). p. 1–9. Available online at: https://www.who.int/publications/i/item/home-care-for-patients-with-suspected-novel-coronavirus-(ncov)-infection-presenting-with-mild-symptoms-and-management-of-contacts (accessed May 24, 2022).

[24] Karaliuniene R Nagendrappa S Jatchavala C Ojeahere MI Ullah I Bytyçi DG. Support the frontliners-good initiatives during the COVID-19 pandemic for healthcare workers across the world: is this what we really need? B J Psych Int. (2022) 1–4. 10.1192/bji.2022.632589718

[25] Tonin L Lacerda MR Caceres NT de G Hermann AP. Recommendations in covid-19 times: a view for home care. Revista Brasileira de Enfermagem, (2020) 73(Suppl 2):e20200310. 10.1590/0034-7167-2020-0310 32609252

[26] Health Human Rights Resource Guide. What is patient care?–Health and Human Rights Resource Guide. (2014). Available online at: https://www.hhrguide.org/2014/02/20/what-is-patient-care/ (accessed May 24, 2022).

[27] Labrague LJ de los Santos JAA Fronda DC. Factors associated with missed nursing care and nurse-assessed quality of care during the COVID-19 pandemic. J Nurs Manag. (2022) 30:62–70. 10.1111/jonm.13483 34590383 PMC8646803

[28] Bergmann M Wagner M. The impact of COVID-19 on informal caregiving and care receiving across europe during the first phase of the pandemic. Front Public Health. (2021) 9:590. 10.3389/fpubh.2021.673874 34222177 PMC8242257

[29] Vadivel R Shoib S El Halabi S El Hayek S Essam L Gashi Bytyçi D. Mental health in the post-COVID-19 era: challenges and the way forward. Gen Psychiatr. (2021) 34:100424. 10.1136/gpsych-2020-100424 33644689 PMC7875255

[30] Jin J-M Bai P He W Wu F Liu F Han DM. Gender Differences in Patients With COVID-19: Focus on Severity and Mortality. Front Public Health. (2020) 8:152. 10.3389/fpubh.2020.00152 32411652 PMC7201103

